# HBx Promotes Liver Cancer Cells to Escape NK‐92 Cell Attack by Mediating ADAM10 to Enzyme Cut MICA/B Shedding From Cancer Cell Membrane

**DOI:** 10.1111/jcmm.71081

**Published:** 2026-03-06

**Authors:** Kailin Huang, Qiushi Yin, Xueqin Wu, Kun Liu, Bo Lin, Wei Li, Yinglian Pan, Mingyue Zhu, Mengsen Li

**Affiliations:** ^1^ Key Laboratory of Tropical Translational Medicine, Ministry of Education Hainan Medical University Haikou Hainan P. R. China; ^2^ Hainan Provincial Key Laboratory of Carcinogenesis and Intervention Hainan Medical University Haikou Hainan P. R. China; ^3^ Department of Medical Oncology, First Affiliated Hospital Hainan Medical University Haikou Hainan P. R. China; ^4^ Department of Medical Oncology, Second Affiliated Hospital Hainan Medical University Haikou Hainan P. R. China

**Keywords:** ADAM10, hepatitis B virus X protein (HBx), immunotherapy, MICA/B, NK‐92 cells

## Abstract

MICA/B shedding from the membrane of cancer cells can inhibit natural killer (NK) cells from attacking hepatocellular carcinoma (HCC). This study explored the role of HBx in mediating MICA/B shedding. The expression of HBx, MICA/B and HIF‐1α in HBV‐infected HCC was analysed using bioinformatics, and the localization of these proteins in tissues was verified using immunohistochemistry and immunofluorescence. HBx‐related signalling pathways were screened using RNA sequencing and KEGG pathway enrichment analyses. The expression of ADAM10 and MICA/B was detected by Western blotting, and the dynamic changes of MICA/B in the membrane and supernatant were evaluated by flow cytometry and ELISA. The HIF‐1α inhibitor (LW‐6) and ADAM10 inhibitor (GI254023X) were used to treat the HCC cells. The killing effect of NK‐92 cells on HCC cells was evaluated using lactate dehydrogenase release, cytotoxicity assays, clone formation and live‐cell imaging, and the secretion levels of IFN‐γ, IL‐2 and IL‐10 were measured. These results indicated that HBx, MICA/B and HIF‐1α were highly expressed in HBV‐infected HCC tissues. HBx promotes shedding of MICA/B from HCC cell membranes by upregulating the activity of ADAM10. LW‐6 reversed the induction effect of HBx on ADAM10 and GI254023X significantly restored MICA/B levels on the membrane surface of HCC cells. Overexpression of HBx increases the resistance of HCC cells to NK‐92 cells and inhibits the secretion of IFN‐γ, IL‐2 and IL‐10. In conclusion, HBx regulates the expression of ADAM10 by activating the HIF‐1α signalling pathway. ADAM10 cuts MICA/B shedding from the membrane surface of HCC cells, resulting in escape attack by NK‐92 cells.

**Abbreviations**
ADAM10: A disintegrin and metalloproteases 10
AKT: protein kinase B
ELISA: enzyme‐linked immunosorbent assay
GO: gene ontology
HBV: hepatitis B virus
HBx: hepatitis B virus X protein
HCC: hepatocellular carcinoma
HIF‐1α: hypoxia‐induced factor‐1α
IFN‐γ: interferon‐γ
IL‐10: interleukin‐10
IL‐2: interleukin‐2
KEGG: Kyoto Encyclopedia of Genes and Genomes
MICA/B: major histocompatibility complex class I chain‐related proteins A and B
mTOR: mammalian target of rapamycin
NK‐92: Natural killer‐92
NKG2D: Natural killer group 2 member D
PI3K: Phosphatidylinosito 3‐kinase
sMICA/B: soluble MICA/B
TCGA: The Cancer Genome Atlas

## 1 Introduction

Hepatocellular carcinoma (HCC) is the most common primary liver malignancy and the third leading cause of tumour‐related deaths worldwide. Its pathogenesis is related to many factors, among which hepatitis B virus (HBV) infection is one of the main causes of chronic liver diseases such as cirrhosis and HCC [1]. At present, the treatment of liver cancer mainly includes surgical resection, liver transplantation, local ablation, chemotherapy, radiotherapy and emerging immunotherapy [2, 3, 4, 5]. However, despite the continuous development of treatment methods, the overall prognosis of liver cancer patients remains poor and the recurrence rate is high, which is largely due to the fact that HCC cells have multiple mechanisms to evade surveillance and attack by the immune system [6, 7].

The HBV genome consists of four open reading frames (ORFs), X, C, S and P, encoding HBx, HBc protein, HBs antigen, HBe antigen and DNA polymerase. These HBV proteins form and assemble into HBV particles and are involved in regulating the expression of viral and host genes [8, 9, 10]. HBx is a multifunctional regulator of gene transcription, signal transduction, cell cycle progression and epigenetic modification [11]. Previous evidence has shown that HBx can stimulate the expression of alpha‐fetoprotein (AFP) to activate the PI3K/AKT/mTOR signalling pathway in hepatocytes and promote carcinogenesis [12]; however, the role of HBx in immune escape remains unclear.

Major histocompatibility complex (MHC) class I chain‐related molecules A and B (MICA/B) are important ligands of the natural killer (NK) group 2D (NKG2D) and are widely expressed on the surface of various tumour cells, including liver cancer cells [13]. Under normal circumstances, MICA/B molecules on the cancer cell surface can bind to NKG2D on the surface of NK cells, transmit activation signals and activate NK cells to kill cancer cells [14]. Therefore, MICA/B molecules play a crucial role in immune recognition and activation of cancer cells and NK cells. However, cancer cells often regulate the expression and distribution of MICA/B molecules to weaken the capacity of NK cells to recognise and attack cancer cells. Studies have shown that HBx may affect the interaction between cancer cells and the immune system in various ways [14]. However, the mechanism by which HBx regulates the distribution of MICA/B on the cell membranes of HCC cells, thus affecting the attack of NK cells on liver cancer cells, has not been fully clarified.

A disintegrin metalloproteinase 10 (ADAM10), also known as CDw156 or CD156c, is encoded by ADAM10. It is produced in the form of a non‐active proenzyme and its mature extracellular structures include metalloproteinases, protein degradins and cysteine‐enrichment domains. The cysteine‐enriched domain is in contact with the metalloproteinase domain, partially covers the active site, and exists in a self‐inhibiting conformation [15]. As a shedding enzyme, it can cleave many proteins, such as TNF‐α, E‐cadherin, CXCL16, angiotensin converting enzyme, etc., by cutting these surface proteins, releasing their extracellular domain, and then regulating relevant signalling pathways. Previous studies have indicated that ADAM10 affects related signalling pathways by cutting membrane protein substrates, thereby promoting cancer cell proliferation, invasion and metastasis [16]. ADAM10 can simultaneously cleave the ligands of immune checkpoint molecules [17]. Hypoxia‐inducible factor 1 (HIF‐1) is composed of two subunits: HIF‐1α and HIF‐1β. HIF‐1α binds to hypoxia response elements (HREs) through HIF‐1β and various coenzyme factors to activate gene transcription. HIF‐1α is the active subunit of HIF‐1 and plays an important role in this signalling pathway [18]. HIF‐1α is a key regulator of metabolic reprogramming in hypoxic cancer cells [19]. Studies have shown that hypoxia in cancer cells increases the resistance of tumour cells to IL‐2/peripheral blood lymphocyte (PBL)‐mediated lysis by stimulating ADAM10 expression, thereby reducing the level of cell membrane MHC molecule A (MICA) [20]. The HIF‐1α signalling pathway plays an important role in angiogenesis, cancer cell proliferation and metastasis, and HIF‐1α is a key effector protein in this signalling pathway with a wide range of target gene profiles [21]. At present, the regulatory mechanism of HIF‐1α and its role in liver cancer warrant further investigation. In this study, we examined the expression of MICA/B in HCC cells and the cutting effect of ADAM10 on the cell membrane MICA/B, and explored the influence of MICA/B on the escape of HCC cells from NK cell attack to provide a new strategy for liver cancer immunotherapy.

## 2 Materials and Methods

### 2.1 Clinical Specimen Collection

Liver cancer tissues and corresponding paracancerous tissues were collected from 30 HBV‐infected HCC patients at the First Affiliated Hospital and the Second Affiliated Hospital of Hainan Medical University from October 2022 to December 2023. Paracancerous tissues were defined as those ≥ 2 cm from the edge of cancerous lesions. Of the 30 patients, 14 were male and 16 were female. The age range was 32–64 years with a mean age of 45.0 years. All enrolled patients underwent radical surgery without any other treatment, the patient's tumour radius is < 3 cm, and the patient's HBV and hepatitis B surface antigen are positive. All patients were followed‐up. About 1.0 × 1.0 × 1.0 cm tissue sample was fixed in 4% paraformaldehyde and then made into tissue chip wax blocks using special instruments. The research protocol was approved by the Ethics Committee of the First Affiliated Hospital of Hainan Medical University, the Second Affiliated Hospital of Hainan Medical University and the Scientific Research Ethics Committee of Hainan Medical University (Ethical Licence number: LW2019312 and 2023‐KYS‐161. This study involved the detection of protein expression in human liver cancer samples, strictly in accordance with the Declaration of Helsinki. The research content and process of the project followed international and national ethical requirements for biomedical research). Written informed consent was obtained from all participants.

### 2.2 Bioinformatics Analysis

We downloaded the required data from The Cancer Genome Atlas (TCGA) and analysed the correlation between HBx and MICA/B in patients with liver cancer caused by HBV infection. The results obtained by RNA‐seq were screened for differentially expressed genes using the R software (4.2.1), and a volcano map was drawn. KEGG enrichment analysis was performed using the R software and visualised using a bubble map.

### 2.3 Immunohistochemical Staining

Immunohistochemical staining was performed on 30 tumour specimens. After dewaxing and antigen repair, the slides were sealed with 3% hydrogen peroxide for 10 min and treated with rabbit anti‐HBx (Cat. #ab223487) and rabbit anti‐MICA/B (Cat. #ab224702) (Abcam Biotech Company, Cambridge, UK) and rabbit anti‐HIF‐1α (Cat. #20960‐1‐AP) overnight at 4°C. After washing, the slices were incubated with Merck‐Calbiochem at room temperature for 60 min and then stained with 3,3‐diaminobenzidine chromogen solution in 3,3‐diaminobenzidine buffer substrate (Merck Chemicals). Sections were visualised using 3,3‐diaminobenzidine and restained with haematoxylin. All the sections were observed under a microscope (ZEISS, Germany).

### 2.4 Extraction and Preparation of Tissue Protein Samples

The 30 tumour specimens fresh tissue blocks were quickly placed in pre‐cooled normal saline, rinsed several times, washed the blood stains, dried the liquid on the surface of the tissue with filter paper, and then the tissue was cut into small pieces. Prepare multiple Eppendorf (EP) tubes, weigh and mark them, place the tissue blocks into EP tubes, weigh them, and add the lysate at a ratio of 100–200 μL lysate per 20 mg of tissue. The homogenate was placed in an ice bath with a tissue grinder for 1–2 min until it was fully cracked. Centrifuge at 10,000–14,000 *g* for 3–5 min, and take the supernatant for follow‐up experiments.

### 2.5 Cell Line

Human HCC cell lines HepG2 and PLC/PRF/5, purchased from Wuhan Punocai Life Technology Co. Ltd. (Wuhan, China), were maintained in Dulbecco's modified Eagle's medium (DMEM) and Minimum Essential Medium (MEM) containing 10% fetal bovine serum and 100 μg/mL penicillin at 37°C and 5% CO_2_. NK‐92 cells purchased from Shanghai Zeye Biotechnology Co. Ltd. (Shanghai, China), NK‐92 cells were derived from peripheral blood mononuclear cells of a 50‐year‐old Caucasian male patient with aggressive non‐Hodgkin lymphoma. The cell line was established in 1992 and is an interleukin‐2 (IL‐2)‐dependent natural killer (NK) cell line. The NK‐92 cells were suspended and cultured using special medium and half liquid exchange methods.

### 2.6 Generation and Screening of Stable HBx Expressed Cell Lines

HepG2 and PLC/PRF/5 cells were prepared in complete culture medium at a controlled concentration of 1 × 10^4^ cells/mL to 5 × 10^4^ cells/mL and inoculated into a six‐well plate. The cells were cultured overnight at 37°C in a 5% CO_2_ incubator until the cell density reached 70%. The cell supernatant was discarded and the cells were washed twice with PBS. Then 1 mL of fresh medium was added, and HitransG P virus infection reagent was added at a ratio of 25:1. The virus volume (μL) was calculated as (multiplicity of infection (MOI) × cell number)/virus titre (TU/mL) × 1000. After mixing the cells well, they were placed in an incubator for further culture for 6 h, the cell supernatant was discarded, washed twice with phosphate‐buffered saline (PBS), and 5 mL of fresh medium was added for further culture. After 72 h of infection, puromycin medium was prepared according to the concentration of purinomycin found in the pre‐experiment for screening. Screening was stopped when all cells in the blank group were observed to have died, then 1/2 of the screening concentration was maintained to continue cell culture, and the transfected cells were screened and expanded.

### 2.7 Western Blotting

The HCC cells were washed twice with pre‐cooled phosphate‐buffered saline (PBS) buffer and then lysed with an affinity lysate in an ice bath for 15 min. The cells were collected in a 15 mL centrifuge tube and centrifuged at 12 000 *g* for 5 min. The supernatant was collected, and total protein was quantified using the bicinchoninic acid assay (BCA) method. Membrane proteins were extracted using a cell membrane protein extraction kit, and the supernatants were collected and quantified using the BCA method. Samples (10 μg total protein/lane) and (20 μg membrane protein/lane) from each experimental group were subjected to sodium dodecyl sulphate‐polyacrylamide gel electrophoresis (SDS‐PAGE) and electrotransfer. The membrane was blocked with a rapid sealing solution for 20 min, incubated with the corresponding primary antibody overnight, and then incubated with the secondary antibody for 1 h. Subsequently, a chemiluminescence imager (Tanon 5200; Shanghai Tanon Scientific and Technical Corporation, Shanghai, China) was used to scan the results and analyse the grey values of each lane.

### 2.8 Laser Confocal Microscope Observation

HCC cells in a good growth state were selected for digestion and diluted into a 3 × 10^4^ cells/mL suspension; 300 μL was sucked out and added to the groove of sterile glass substrate, placed in an incubator for culture until they completely adhered to the wall, and the shape was developed, cleaned with PBS buffer, and then fixed with 4% paraformaldehyde 500 μL in drops for 30 min. After fixation, the cells were rinsed twice with PBS and 500 μL of goat serum was added to a glass substrate culture dish for closure (no permeability) for 30 min. After complete washing with PBS, 200 μL (1:100) of diluted primary antibody (rabbit anti‐MICA/B [Cat. #ab224702] [Abcam Biotech Company, Cambridge, UK]) was added to a glass substrate dish and incubated at 4°C overnight. The next day, the primary antibody was recovered, the cells were moistened, the secondary antibody of diluted CoraLite 594 with fluorescence labeling was added (1:200), and the cells were incubated at 37°C in the dark for 1 h, and then moistened, each dish was incubated with 100 μL of anti‐fluorescence quenching sealing tablets (containing DAPI staining solution), and the tablets were observed and photographed using a laser confocal microscope (ZEISS, LSM 800 with Airyscan, Germany) for 20 min.

### 2.9 Enzyme‐Linked Immunosorbent Assay (ELISA)

The ELISA method was used to detect the soluble MICA/B (sMICA/B) content in the supernatant of HCC cells. All experimental groups were divided into 2 h at room temperature equilibrium, and a blank control hole was set up without adding any liquid. The corresponding calibrator (50 μL), sample diluent (50 μL), serum, quality control product (50 μL) and biotin antibody (100 μL) were then added to each well and incubated at 37°C for 60 min. The plate was washed five times, dried, 100 μL of HRP‐labelled avidin was added, and incubated at 37°C for 20 min. The plate was washed five times, thoroughly mixed with substrate A and B at a 1:1 volume, added the substrate mixture of 100 μL to all wells, covered with a sealing plate film and incubated at 37°C for colour development for 15 min. A termination solution (50 μL) was added to all wells and the absorbance (OD value) of each well was measured at 450 nm.

### 2.10 The Killing Effect Was Observed by High Throughput Imaging of Intelligent Living Cells Instrument

HCC and NK‐92 cells in the logarithmic growth phase were placed in a 6‐well plate at a 2:1 ratio and both were suspended by shaking the cells. The 6‐well plate was placed in an intelligent living cell high‐throughput imaging instrument (Thermo Fisher EVOS M7000 Intelligent living cell imaging analysis system), and pictures were taken every 15 min for 15 h to record the entire process of NK‐92 cells killing a total of the liver cancer cells.

### 2.11 Plate Clone Formation Experiment

HCC and NK‐92 cells with good growth status were selected, and the number of cells inoculated in each well was inoculated into a 6‐well plate at a ratio of 4:1 according to the cell proliferation rate and growth characteristics of nuclear cells, which were mixed evenly and then cultured in an incubator. When the clones were visible to the naked eye, the culture medium was removed and completely cleaned with 1 × PBS, and 2 mL of 4% paraformaldehyde was added to each well to fix the cells. The paraformaldehyde was removed, and crystal violet was added for dyeing. The number of colonies was counted under a microscope.

### 2.12 Statistical Analysis

Image J software was used to calculate the grey value of Western blotting lane and the cell‐positive cell ratio of IHC images, and the statistical results were expressed in the form of mean ± standard error (SEM). Statistical analysis was performed and charts were drawn by GraphPad Prism 10.0 software. For three independent samples, a *t*‐test was conducted, and comparisons involving three or more groups used one‐way analysis of variance (ANOVA) statistical analysis of the data. *p* < 0.05 was considered statistically significant.

## 3 Results

### 3.1 Expression and Gene Correlation Analysis of HBx, MICA/B and HIF‐1α in Biological Information Database

**FIGURE 1:**
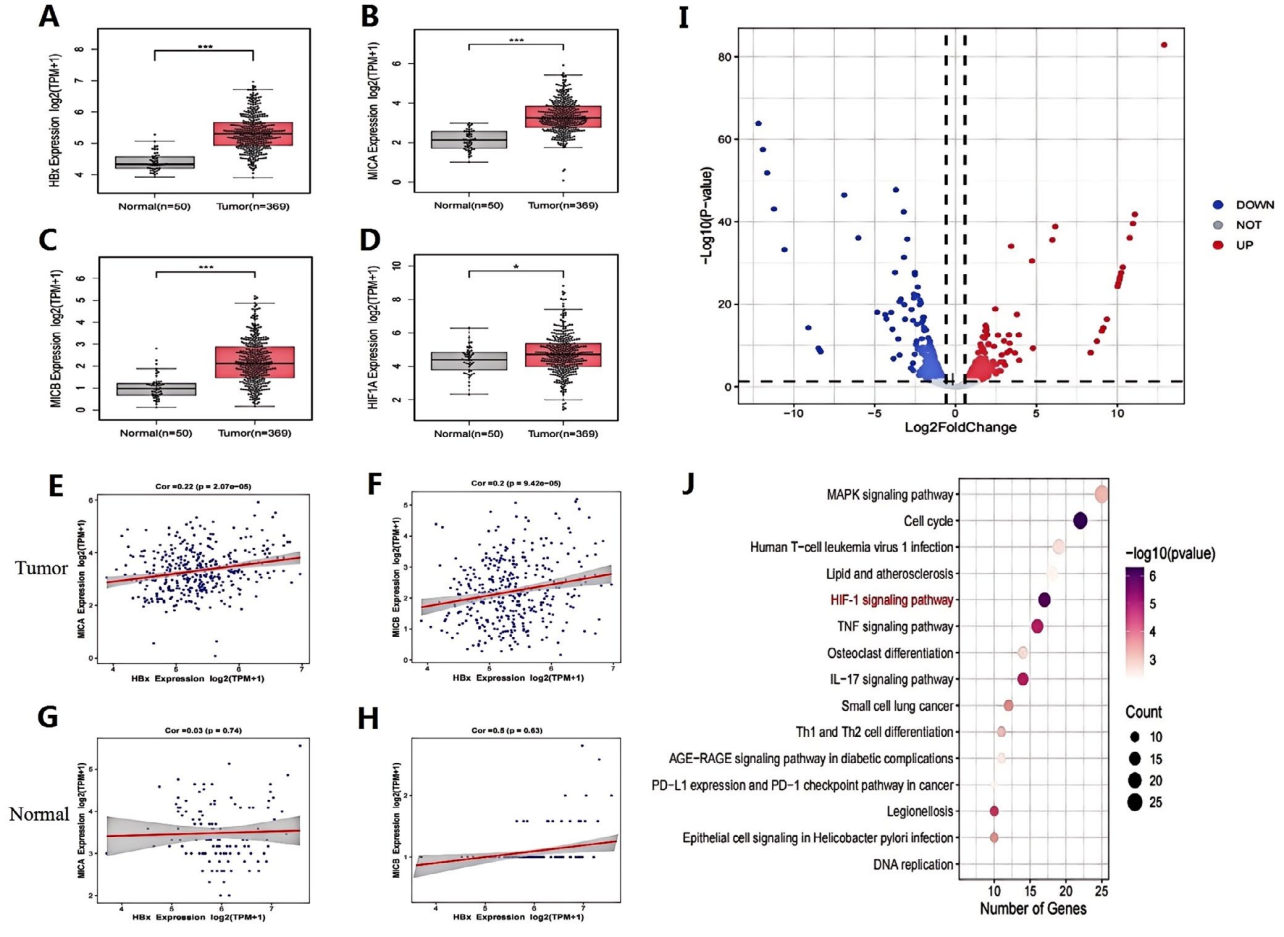
Expression of HBx, MICA/B and HIF‐1α in database and gene correlation analysis. (A–D) The expression levels of HBx, MICA/B and HIF‐1α in HCC tissues and adjacent normal (paracancerous) tissues were analysed. (E–H) Correlation analysis was performed between HBx and MICA/B. (I) Volcano maps showing differentially expressed genes from RNA‐seq sequencing data. (J) KEGG enrichment analysis. Mean ± standard deviation was used for statistical data and evaluated by the *t*‐test. **p < 0.01, ***p < 0.001.

HCC RNA‐seq data were downloaded from the TCGA database to analyse the expression levels of HBx, MICA/B and HIF‐1α in HCC tissues and adjacent normal (paracancerous) liver tissues [22]. This study's results showed that the expression levels of HBx, MICA, MICB and HIF‐1α in HCC tissues were higher than those in normal liver tissues (Figure 1A–D). The scatter plot results of gene correlation showed that HBx was positively correlated with MICA (*r* = 0.22, *p* = 2.07e‐05) and MICB (*r* = 0.22, *p* = 2.07e‐05) in the HCC tissues. In normal tissues, HBx did not correlate with MICA or MICB (Figure 1E–H). RNA‐seq was performed on pLV‐HBx and pLV‐NC, and the results were analysed using R language [23]. The results showed that after overexpression of HBx, the expression of 485 genes was significantly upregulated, and that of 493 genes was significantly decreased (Figure 1I). Subsequently, the results of RNA‐seq sequencing were analysed for KEGG enrichment [24], and the results showed that the enrichment pathways mainly included the mitogen‐activated protein kinase (MAPK), HIF‐1 and tumour necrosis factor (TNF) signalling pathways (Figure 1J).

### 3.2 HBx, MICA/B and HIF‐1α Are Highly Expressed in HBV‐Related Liver Cancer Tissues

**FIGURE 2:**
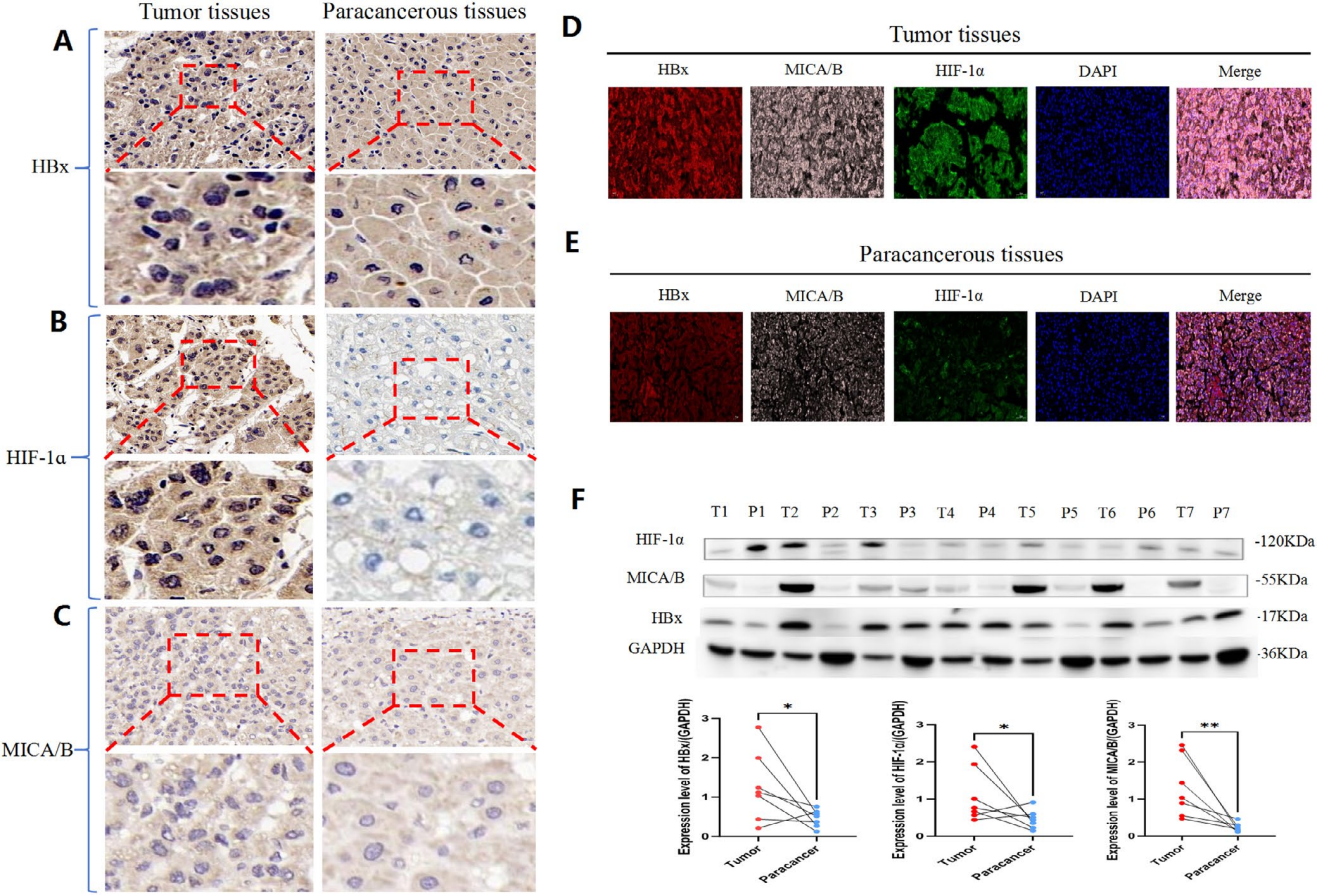
The influence of HBV on the expression of HBx, MICA/B and HIF‐1α in liver cancer tissues. (A–C) The expression levels of HBx, MICA/B and HIF‐1α in liver cancer tissues and adjacent normal (paracancerous) liver tissues were detected by immunohistochemistry. (D, E) Immunofluorescence assay was used to observe the expression and localization of HBx, MICA/B and HIF‐1α in liver cancer tissues and paracancerous tissues. (F) Western blotting was used to detect the expression levels of HBx, MICA/B and HIF‐1α in liver cancer tissues and paracancerous tissues. Mean ± standard deviation was used for statistical data and evaluated by the *t*‐test. *p < 0.01, **p < 0.001.

In this study, cancer tissues and corresponding adjacent (paracancerous) tissue samples of 30 HCC patients infected with HBV were prepared into tissue chips, the expression of HBx, MICA/B and HIF‐1α was detected by immunohistochemistry, and the density of positive cells in the sections was analysed. The results showed that the expression of HBx, MICA/B and HIF‐1α was significantly higher in cancer tissues than in paracancerous tissues (Figure 2A–C). Immunofluorescence was used to observe the expression and localization of HBx, MICA/B and HIF‐1α in liver cancer and corresponding paracancerous tissues. The results showed that HBx was mainly located in the cytoplasm and in small amounts in the nucleus, MICA/B was mainly located in the cytoplasm, and HIF‐1α was mainly located in the cytoplasm and nucleus. The fluorescence intensity of liver cancer tissues was more obvious than that of paracancerous tissues (Figure 2D,E). Western blotting analysis showed that the expression of HIF‐1α, MICA/B and HBx in cancer tissues was significantly higher than that in paracancerous tissues (Figure 2F). These results indicated that HBx, MICA/B and HIF‐1α were highly expressed in HBV‐infected HCC tissues.

### 3.3 HBx Expressed or Interfered in HCC Cell Lines Was Successfully Constructed

HCC cell lines with stable overexpression of HBx and interference with HBx were constructed, and a no‐load control group was simultaneously established. In the same position, photos under white light and green fluorescence were compared, and the transfection efficiency of the cells was observed to be 90% (Figure S1A,C). Western blotting analysis results showed that compared with the control and no‐load groups, HBx protein expression levels in the HepG2‐HBx group were significantly increased (Figure S1B), while that in the PLC/PRF/5‐shHBx group was significantly decreased (Figure S1D). These results indicated that a stable cell line was successfully constructed.

### 3.4 HBx Reduced the Content of MICA/B on the Membrane Surface of HCC Cells

**FIGURE 3:**
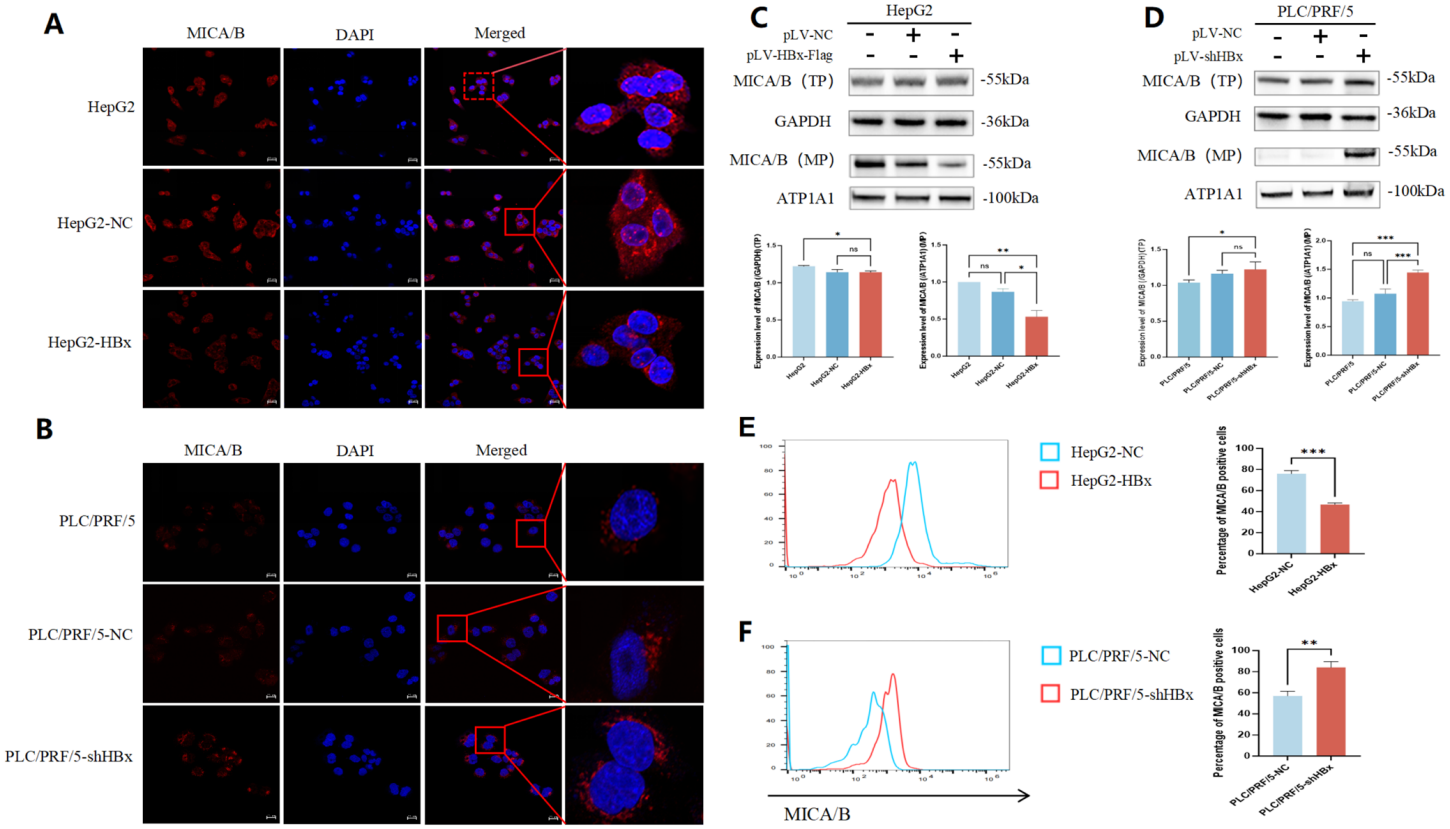
The effect of HBx in the expression of MICA/B on the membrane of liver cancer cell. (A) The expression and localization of MICA/B in HepG2 (HepG2‐NC, HepG2‐HBx) cells was observed by laser confocal microscopy. CoraLite594 (stained MICA/B) cells fluoresce red (red), DAPI stain (blue) and the images were merged. (B) The expression and localization of MICA/B in PLC/PRF/5 (PLC/PRF/5‐NC, PLC/PRF/5‐shHBx) cells was observed by laser confocal microscopy. CoraLite594 (MICA/B) in cells fluoresces red (red), DAPI staining (blue), and images were merged. (C, D) The expression levels of MICA/B total protein (TP) and membrane protein (MP) were detected by Western blotting, ATP1A1 was used as the internal control; the bar chart below is the statistics of the grey scan values of the protein bands. (E, F) Flow cytometry was used to detect the expression of MICA/B in HepG2‐NC cells and HepG2‐HBx cells as well as PLC/PRF/5 and PLC/PRF/5‐shHBX cells, and the proportion of MICA/B positive cells, the bar chart on the right is a statistical analysis chart. MP, membrane protein; TP, total protein; the length of the scale is 200 μm, and mean ± standard deviation was used for statistical data and evaluated by the *t*‐test. ns means *p* > 0.05, *p < 0.05, **p < 0.01, **p < 0.001. All experimental results must be repeated at least three times.

Laser confocal microscopy was used to observe the localization of MICA/B in HepG2 and PLC/PRF/5 cells, and the results showed that MICA/B was expressed in both the cytoplasm and cell membrane (Figure 3A,B). Furthermore, Western blotting analysis showed that changes in HBx expression had no significant influence on the expression level of total MICA/B protein in HCC cells (Figure 3C). However, the expression level of the membrane protein MICA/B was significantly decreased in HepG2 cells after HBx overexpression and significantly increased in PLC/PRF/5 cells after interference with HBx expression (Figure 3D). Flow cytometry results also verified these results (Figure 3E,F). These results indicated that overexpression of HBx significantly reduced the content of MICA/B on the membrane surface of HCC cells; however, interference with the expression of HBx significantly increased the content of MICA/B on the membrane surface of liver cancer cells.

### 3.5 HBx Increased Content of Soluble MICA/B (sMICA/B) by Stimulating ADAM10 Expression

**FIGURE 4:**
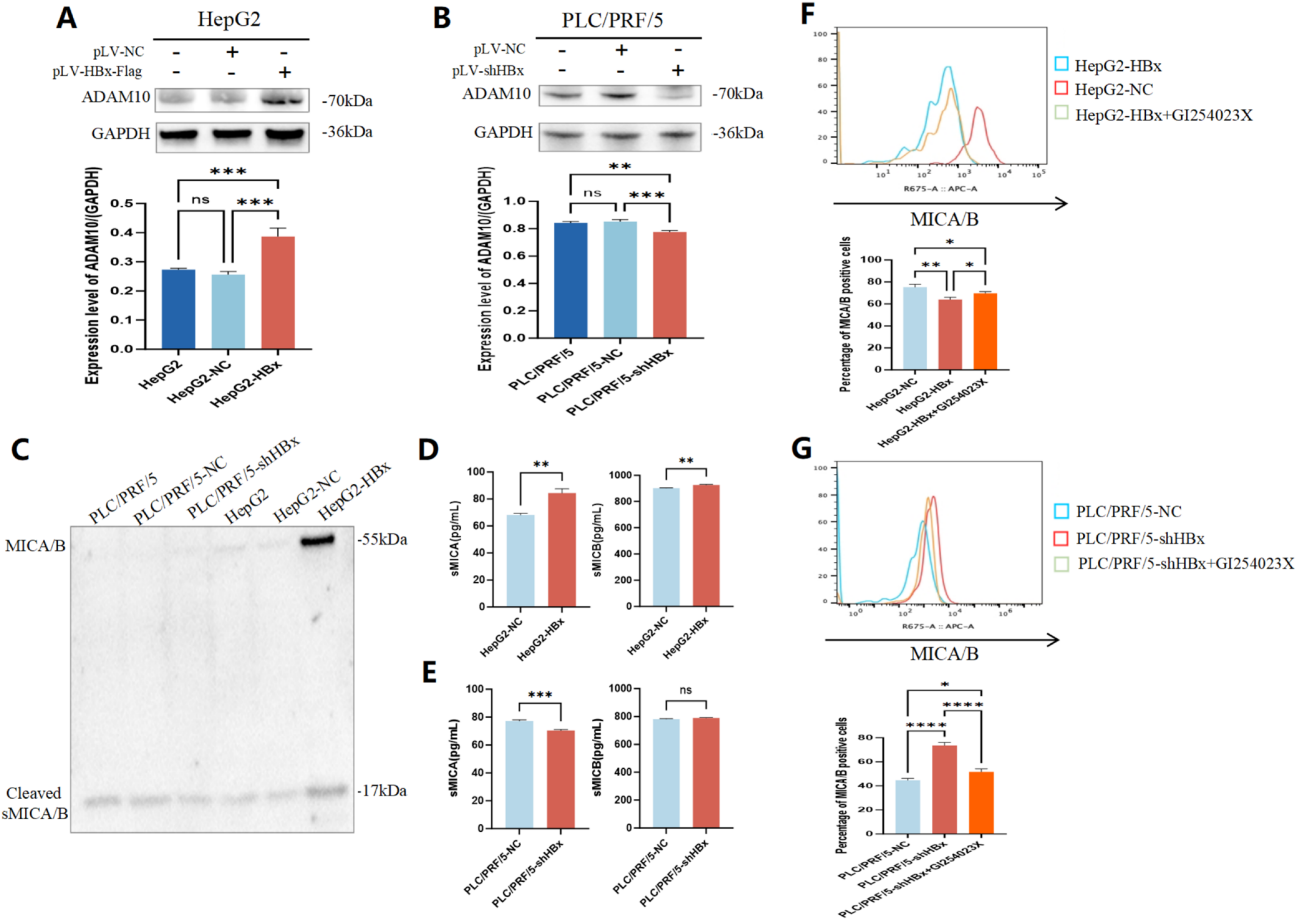
HBx regulated the expression of ADAM10 to influence the content of soluble MICA/B (sMICA/B) in HCC cells. (A, B) Western blotting was applied to detect the influence of HBx on the expression of MICA/B in HCC cells while overexpression or interference with HBx, the bar chart below is a statistical map of the grey value scanning of protein bands. (C) The expression of sMICA/B in the supernatant of HepG2, HepG2‐NC, HepG2‐HBx cells and PLC/PRF/5, PLC/PRF/5‐NC, PLC/PRF/5‐shHBx cells was detected by Western blotting. (D) The content of sMICA/B in HepG2‐NC and HepG2‐HBx cells was detected by ELISA. (E) The content of sMICA/B in PLC/PRF/5‐NC and PLC/PRF/5‐shHBx cells was detected by ELISA. (F, G) Flow cytometry was used to detect the influence of HBx on the content of MICA/B in cellular membrane of liver cancer, the bar chart below is a statistical analysis of the percentage of MICA/B positive on the cellular membrane of liver cancer. ns means *p* > 0.05; *p < 0.05, **p < 0.01, ***p < 0.001; ****p < 0.0001. These results are represent of three repeated experiments.

According to previous studies, ADAM10 is an important cleavage transmembrane enzyme. As a cutting enzyme, ADAM10 affects relevant signalling pathways and cell biological behaviours by cleaving off the membrane protein substrates [25]. Therefore, we speculated whether HBx plays a role in cutting MICA/B. To explore the mechanism by which HBx regulates MICA/B content on the membrane of HCC cells, we first used Western blotting to analyse the changes in ADAM10 expression mediated by HBx. The results showed that the expression level of ADAM10 was significantly increased when HBx was overexpressed (Figure 4A); however, ADAM10 expression was decreased when HBx was knocked down (Figure 4B). The supernatants of the two HCC cell lines were then extracted for Western blotting analysis, and the results showed that MICA/B was cut into two parts with two different molecular weights (50 and 17 kDa) (Figure 4C). Further quantitative analysis by ELISA showed that the concentration of soluble MICA/B (sMICA/B) in the HBx overexpression group was significantly higher than that in the control group (Figure 4D), and the concentration of sMICA/B in the HBx‐expressing group was lower than that in the control group (Figure 4E). An ADAM10 inhibitor (GI254023X) may reverse the effect of HBx by decreasing MICA/B content in the membranes of liver cancer cells. When the cells were treated with GI254023X, the expression level of MICA/B increased significantly (Figure 4F,G). These results indicated that HBx could promote the expression of ADAM10, thus stimulating the cleavage of MICA/B on the membrane surface of liver cancer cells, resulting in an increase in sMICA/B concentration in the cell supernatant; however, ADAM10 inhibitors could reverse this effect.

### 3.6 HBx Stimulates ADAM10 Expression to Reduce the Content of MICA/B on the Membrane Surface of HCC Cells Through Activating HIF‐1α Signalling Pathway

**FIGURE 5:**
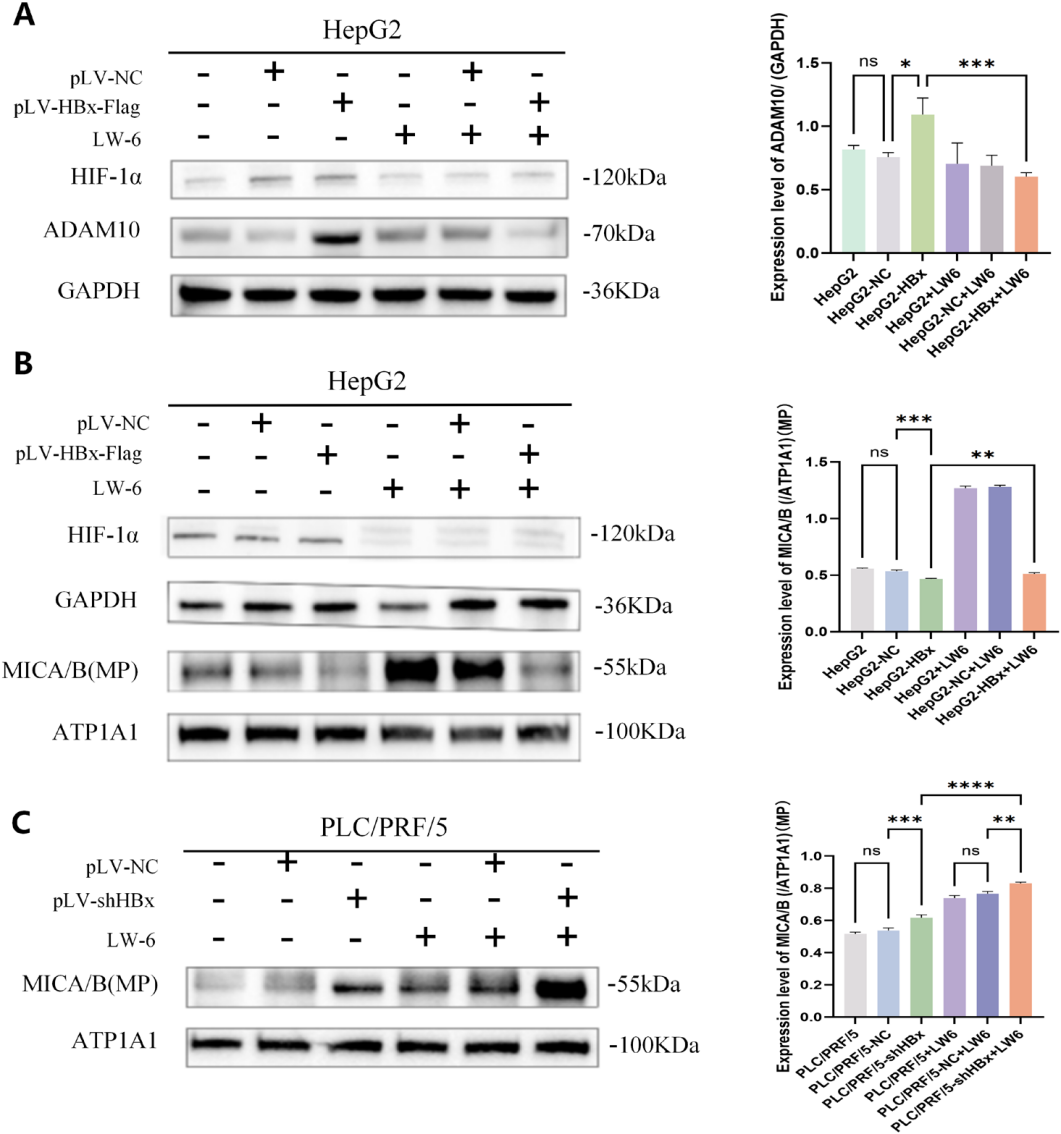
The role of HBx in the expression of ADAM10, HIF‐1α and affect the content of MICA/B on membrane of liver cancer. (A) Western blotting analysis was used to detect the expression changes of ADAM10 and HIF‐1α protein after treated with of HIF‐1α signalling inhibitor (LW6); the bar chart on the right is a statistical graph of protein grey scan values. (B, C) Western blotting was used to analyse the expression changes of MICA/B protein on the cellular membrane of liver cancer cell after LW6 was treated; the bar chart on the right is a statistical graph of protein grey scan values. ns mean *p* > 0.05, **p < 0.05, ***p < 0.001, ****p < 0.0001. The image represents the results of three repetitions.

LW6 is a specific inhibitor of the HIF‐1 signalling pathway [26]. In this study, we used LW6 to treat liver cancer cells, extract proteins, and perform Western blotting experiments. The results showed that the expression of ADAM10 was significantly downregulated after treatment with LW6 in liver cancer cells, and the upregulation effect of HBx on ADAM10 was reversed (Figure 5A). These results implied that HBx regulates ADAM10 expression by activating the HIF‐1α signalling pathway. Western blotting was performed to analyse the changes in membrane MICA/B expression after treatment with LW6. The results showed that after treatment with LW6, the effect of HBx on reducing MICA/B in the HCC cell membrane surface was reversed, and the total MICA/B content increased (Figure 5B,C). These results indicate that HBx promotes the expression of ADAM10 by activating the HIF‐1α signalling pathway. After inhibiting the HIF‐1α signalling pathway, the effect of HBx overexpression on decreasing the HCC cell membrane surface MICA/B was reversed.

### 3.7 HBx Suppresses the Cytotoxicity of NK‐92 Cells to HCC Cells

**FIGURE 6:**
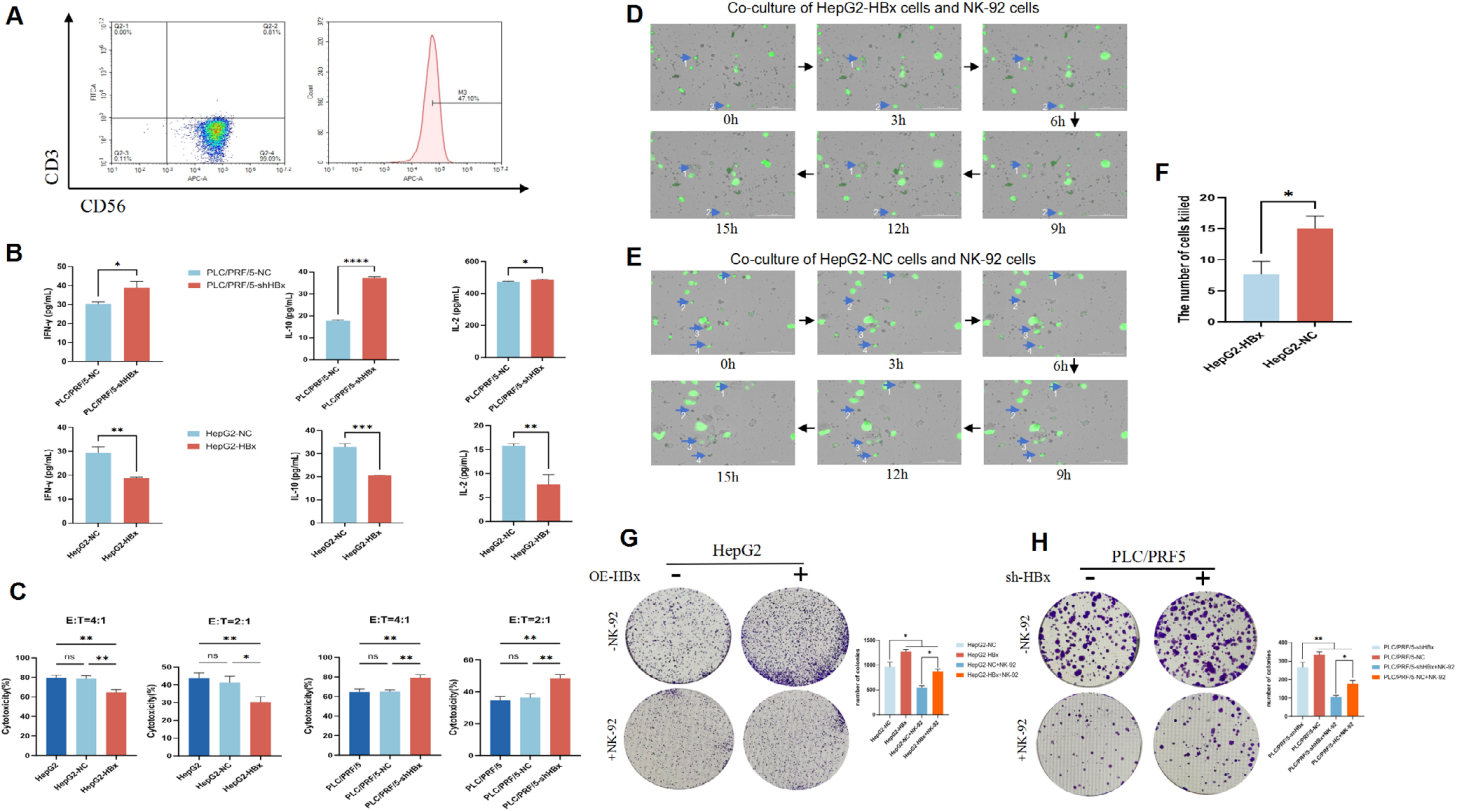
The influence of HBx on the cytotoxicity of NK‐92 cells to liver cancer cells. (A) Expression of CD56 and CD3 in NK‐92 cells was detect by flow cytometer. (B) The contents of cytokines (IFN‐γ, IL‐10, IL‐2) in the cell supernatant were detected by ELISA. (C) Cytotoxicity of NK‐92 cells to HCC cells (HepG2, HepG2‐NC, HepG2‐HBx, PLC/PRF/5, PLC/PRF/5‐NC, PLC/PRF/5‐shHBx); (E) T indicates the ratio of NK‐92 cells to the number of liver cancer cells. (D, E) Dynamic monitoring of NK‐92 cells and HCC cells (HepG2‐HBx, HepG2‐NC) during co‐culture was observed by high throughput intelligent living cells instrument. The white is NK‐92 cells and the green is liver cancer cells. (F) Statistical chart of the number of HCC cells were killed. (G, H) HBx negative and no NK‐92 cells were set as the double negative control group, and the killing effect of NK‐92 cells on liver cancer cells was detected by cloning formation, the bar chart on the right is a statistical plot of the number of cell clones. ns mean *p* > 0.05, *p < 0.05, **p < 0.01, ***p < 0.001, ****p < 0.0001. These results are represent of three repeated experiments.

CD56^+^ and CD3^−^ are classical markers of NK cells [27]. In the present study, we used flow cytometry to detect these markers in the NK‐92 cells. The results showed that NK‐92 cells exhibited a typical NK phenotype (CD56^+^, CD3^−^) (Figure 6A). ELISA results showed that interferon‐γ, interleukin‐10 and interleukin‐2 in the co‐cultured NK‐92 cells with HepG2‐HBx cells were significantly reduced compared to those in the co‐cultured NK‐92 cells with HepG2‐NC group. In contrast, the levels of these cytokines were significantly increased in NK‐92 cells co‐cultured with PLC/PRF/5‐shHBx cells (Figure 6B). We co‐cultured NK‐92 cells with different proportions of HCC cells and conducted a lactate dehydrogenase assay to detect NK‐92 cell‐killing activity. The results showed that the cytotoxicity of NK‐92 cells in the HBx‐overexpressing group (HepG2‐HBx) was significantly lower than that in the control group. The cytotoxicity of NK‐92 cells in PLC/PRF/5‐shHBx cells was higher than that of the control group (Figure 6C). Subsequently, an intelligent live cell high‐throughput imaging analyser was used for the dynamic monitoring and imaging of co‐cultured cells, including dynamic monitoring images taken during the co‐culture of NK‐92 cells with HepG2‐HBx cells (Figure 6D) and dynamic monitoring images taken during the co‐culture of NK‐92 cells with HepG2‐NC cells (Figure 6E). Five fields were randomly selected from each group and the number of HCC cells killed in each field was counted for statistical analysis. Statistical analysis showed that the number of NK‐92 cells was significantly lower in HepG2‐HBx cells than in HepG2‐NC cells (Figure 6F). Dynamic monitoring videos are shown as Videos S1 and S2. Liver cancer cells and liver cancer cells stably transfected with overexpressed HBx (OE‐HepG2) vectors or small hairpin‐interfered HBx (PLC/PRF/5‐shHBx) vectors were co‐cultured with or without NK‐92 cells in a 6‐well plate for 24 h, and then stained with crystal violet to observe the effect of NK‐92 cells on the clonal formation of HCC cells. The results showed that, compared with the control group, OE‐HepG2 cells had more colonies (Figure 6G); however, in the PLC/PRF/5‐shHBx cells, the colony numbers were significantly decreased compared with the control groups (Figure 6H). These results indicated that overexpression of HBx in HepG2 cells inhibited NK‐92 cells from attacking HCC cells; however, NK‐92 cells effectively attacked PLC/PRF/5 cells while interfering with the expression of HBx.

## 4 Discussion

Cancer cell immunity evades recognition and attack by the immune system, leading to the survival, proliferation and metastasis of cancer cells in the body through various mechanisms. Under normal circumstances, the immune system can recognise and eliminate cancer cells, which mainly depends on the recognition of tumour‐associated antigens by immune cells such as effector T cells and NK cells [28, 29, 30]. However, cancer cells have evolved various strategies to evade immune surveillance, such as immune escape, by reducing MICA/B molecules on the cell membrane surface. HBx protein can affect cell cycle regulation and apoptosis and plays a pivotal role in initiating liver cancer formation in the course of chronic active hepatitis and cirrhosis. It has been proved that HBx protein could induce the expression of AFP to activate the PI3K/AKT/mTOR signalling pathway in hepatoma cells [12]. It has been reported that AFP could inhibit the maturation of dendritic cells, thus promoting the downregulation of NKG2D expression by IL‐12 and ultimately enabling HCC cells to evade the immune surveillance of NK cells [31]. NKG2D is an activated receptor on the surface of NK cells [32], whereas MICA/B is a ligand of NKG2D. The MICA/B‐NKG2D pathway is the main pathway that promotes NK cell activation [33]. These research results suggest that it is highly necessary to explore the regulatory role of the MICA/B‐NKG2D pathway in liver cancer cells evading NK cell attack.

In this study, we first analysed the expression levels of HBx, MICA and MICB in HBV‐infected HCC tissues compared to those in paracancerous tissues, and analysed the correlation between HBx, MICA/B and HIF‐1α. Immunohistochemistry results indicated high expression of HBx, MICA/B and HIF‐1α in the HCC tissues. Immunofluorescence and Western blotting were used to observe the localization and expression of HBx, MICA/B and HIF‐1α in these tissues. The results showed that the expression levels of HBx, MICA/B and HIF‐1α were significantly higher in HCC tissues than those in paracancerous tissues. HCC cell lines with stable HBx overexpression and HBx expression were generated. RNA‐seq results showed that there were differential genes after HBx overexpression, and KEGG enrichment analysis suggested some major cellular signalling pathways. Laser confocal microscopy results showed that MICA/B was more localised in the HCC cell membrane, but less expressed in the cytoplasm. While the expression of MICA/B protein was detected by Western blotting, the results showed that the total protein content of MICA/B remained unchanged. However, the content of MICA/B membrane protein significantly decreased after overexpression of HBx and significantly increased after interference with HBx expression in HCC cells, which was also verified by flow cytometry.

**FIGURE 7:**
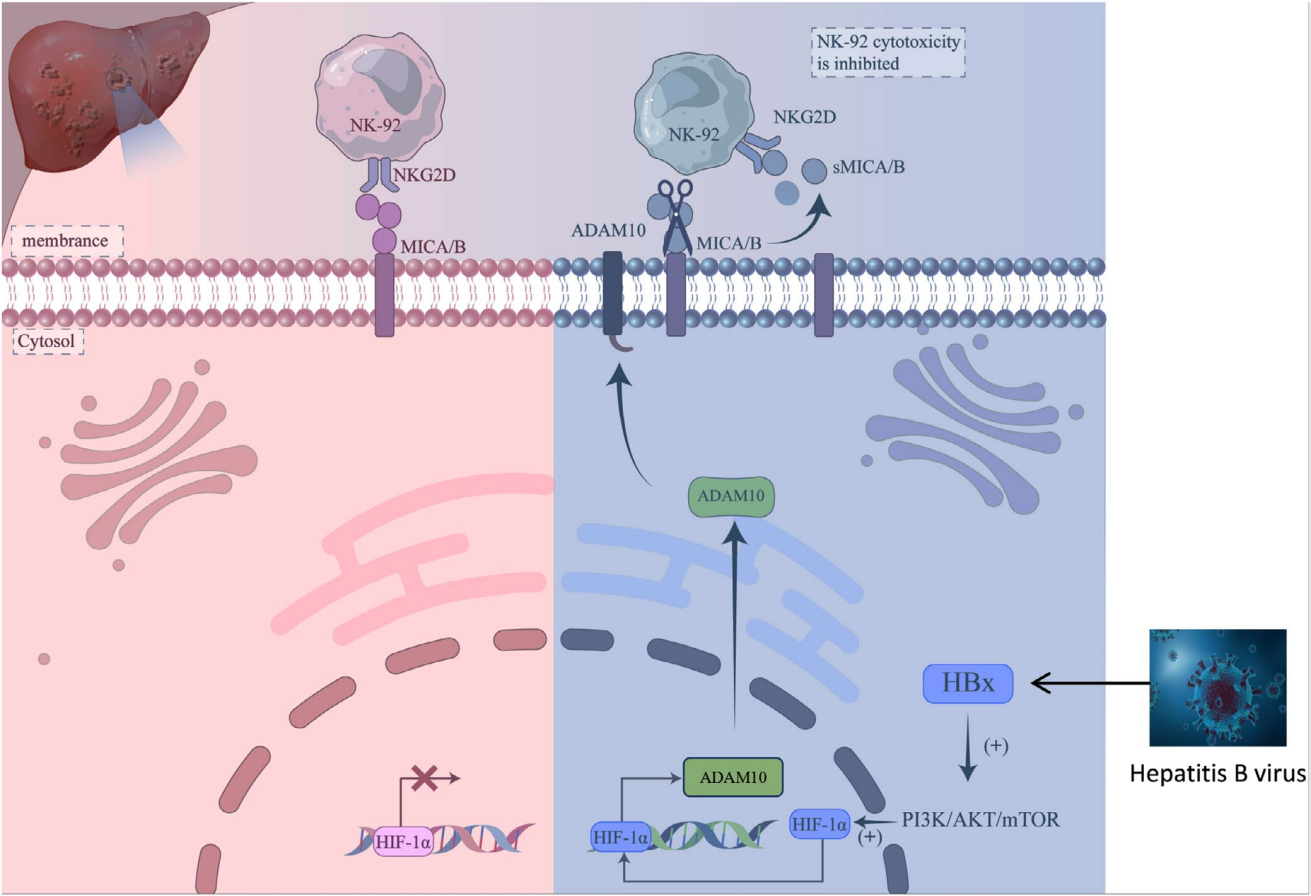
Schematic illustration of HBx‐mediated ADAM10 to cut MICA/B shedding from the membrane of HCC cell leads to escape natural killer cell attack. MICA/B binds to the NKG2D receptor on NK‐92 cells, and NK‐92 cells recognise and kill liver cancer cells. Under HBV infection, HBx regulates the transcription initiation of HIF‐1α by activating PI3K/AKT signalling pathway and promotes the expression of ADAM10. ADAM10 is regulated by HIF‐1α signalling pathway to cut MICA/B shedding from the membrane of HCC cell. After MICA/B is shed from the cellular membrane of liver cancer, on the one hand, the content of MICA/B on the cellular membrane of liver cancer is reduced; on the other hand, the shed MICA/B enters the tumour microenvironment and forms sMICA/B, which binds with NKG2D of NK‐92 cells. By blocking the recognition of MICA/B on the cellular membrane of liver cancer, NKG2D reduces the binding of NK‐92 cells to liver cancer cells, interferes with the attack of NK‐92 cells on liver cancer cells, and causes liver cancer cells to escape immune surveillance.

To explore the mechanism by which HBx regulates MICA/B expression and mediates immune escape, we speculated that HBx protein promotes the expression of ADAM10 by activating the HIF‐1ɑ signalling pathway and that MICA/B is shed from the cell membrane surface under the activation of ADAM10, thus reducing the content of MICA/B on the cell membrane surface of liver cancer cells. While MICA/B is shed from the membrane surface of cancer cells, sMICA/B binding to NKG2D in NK cells leads to the interaction between NKG2D and MICA/B in cancer cells, thereby reducing the recognition of liver cancer cells by NK cells. In the present study, the results showed that overexpression of HBx inhibited the release of cytokines (IFN‐γ, IL‐10 and IL‐2) from HCC cells and reduced the cytotoxicity of NK‐92 cells to liver cancer cells. In addition, the results of the live‐cell high‐throughput experiment showed that the resistance of HCC cells to NK‐92 cytotoxicity was significantly enhanced while overexpressing HBx in HCC cells, and the results of the cloning formation experiment showed that NK‐92 cells had a more significant killing effect on HCC cells, while interfering with HBx expression. These results indicated that the expression level of HBx was negatively correlated with the killing effect of NK‐92 cells on HCC cells. Since HBx can stimulate AFP expression, activate the PI3K/AKT signalling pathway, and promote the expression of HIF‐1α [34, 35, 36, 37, 38], we speculated that HBx may promote HIF‐1α to induce the expression of ADAM10 by activating the PI3K/AKT signalling pathway, resulting in ADAM10 cleavage of MICA/B in the HCC cell membrane. The mechanism by which HBx induces MICA/B shedding in the liver cancer cell membranes is shown in Figure 7. Our study demonstrated that HBx had no significant effect on total MICA/B expression in HCC cells, but could significantly increase MICA/B shedding, suggesting that HBx not only promotes the occurrence and development of HCC, but more importantly, stimulates liver cancer cells to escape the attack of NK cells. This study showed that HBx is an important target molecule for liver cancer immunotherapy.

## 5 Conclusion

This study showed that HBx regulates HIF‐1α transcription initiation and promotes ADAM10 expression. ADAM10 can increase MICA/B shedding from the membrane surface of HCC cells by activating the HIF‐1α signalling pathway, and sMICA/B is shed in the supernatant, thus blocking the recognition of NKG2D by MICA/B on the membrane of HCC cells, reducing the cytotoxicity of NK‐92 cells, and enhancing the resistance of HCC cells to attack by NK‐92 cells, leading to significant inhibition of NK‐92 cell to attack HCC cells. Our findings further prove that HBx plays a critical role in the survival of early stage HCC cells, because HBx can stimulate the expression and activity of ADAM10 to cut MICA/B on the membrane surface of HCC cells, resulting in the inability of NK cells to recognise HCC cells and cause HCC cells to escape immune surveillance. Thus, HBx and MICA/B could be used as target molecules to develop vaccines for HBV‐related HCC immunotherapy.

## Author Contributions

K.H., Q.Y., X.W., K.L., B.L. and W.L. contributed to conceptualization and performed data collection, bioinformatics analysis and immunofluorescence assays. K.H. and X.W. performed immunohistochemical and Western blotting experiments and wrote the original draft. Q.Y. performed ELISA and flow cytometry. M.L. and M.Z. designed and supervised the study, analysed the data and wrote and reviewed the manuscript. All authors have read and approved the final version of the manuscript.

## Funding

This work was supported by the National Natural Science Foundation of China (Nos. 82460602, 82573045, 82060514, 82560459 and 81960519), Natural Science Foundation of Hainan Province (Nos. 824RC517 and 822RC700) and the Hainan Province Science and Technology Special Foundation (No. ZDYF2021SHFZ222), the Research Project of Take off the Proclamation and Leadership of the Hainan Medical University Natural Science Foundation (No. JBGS202106) and Hainan Provincial Association for Science and Technology Program of Youth Science Talent and Academic Innovation (QCXM 201922).

## Ethics Statement

Study approval statement: The study protocol was reviewed and approved by the Ethics Committee of the First Affiliated Hospital of Hainan Medical University, the Second Affiliated Hospital of Hainan Medical University and the Scientific Research Ethics Committee of Hainan Medical University. Approval Ethical Licence Numbers LW2019312 and 2023‐KYS‐161. All procedures performed in this study involving human participants were in accordance with the ethical standards of the institutional and/or national research committee and with the 1964 Helsinki Declaration and its later amendments or comparable ethical standards.

## Consent

Written informed consent was obtained from all the participants.

## Conflicts of Interest

The authors declare no conflicts of interest.

## Supporting information


**Figure S1:** Construct stable cell lines and explore the influence on the biological process of HCC cells. (A) Fluorescence microscopy of pLV‐NC and pLV‐HBx lentiviral cells transfected HepG2 with fluorescence microscopy. (B) Expression of HBx protein in HepG2 cells was detected by western imprinting. (C) Fluorescence expression of pLV‐NC and pLV‐shHBx lentiviral cells transfected with PLC/PRF5 was observed by fluorescence microscopy. (D) Expression of HBx protein in PLC/PRF/5 cells was detected using Western blotting. The error line represents ± standard deviation evaluated using the *t*‐test, and the scale length was 200 μm. ns means *p* > 0.05; *p < 0.05, **p < 0.01, ****p < 0.0001. The scale length was 200 μm. These results are represent of three repeated experiments.

**Videos S1–S2:** High throughput intelligent living cells instrument was applied to observe the attack activity of NK‐92 cells to HepG2 cells. Video S1: HepG2 cells transfected with the control vector and co‐cultured with NK‐92; Video S2: HepG2 cells transfected with HBx overexpression vectors and co‐cultured with NK‐92 cells. The effect of NK‐92 on HepG2 cells was observed for 12 h using a high‐throughput intelligent living‐cell instrument. These results are represent of three repeated experiments.
**Video S1:** http://video.cqdbc.org.cn/mp4/0422/1/.
**Video S2:** http://video.cqdbc.org.cn/mp4/0422/2/.

## Data Availability

The database used for the analysis of the biological information in this study can be accessed online. The data used in this study were obtained from a database: (1) the TCGA database, https://portal.gdc.cancer.gov and (2) the KEGG database, https://www.kegg.jp. If used, the corresponding author may contact the research data generated in this study.
