# Supplementary material for: HBx Promotes Liver Cancer Cells to Escape NK‐92 Cell Attack by Mediating ADAM10 to Enzyme Cut MICA/B Shedding From Cancer Cell Membrane
Source: J Cell Mol Med. 2026 Mar 6;30(5):e71081. doi: 10.1111/jcmm.71081 (PMC12965905; doi:10.1111/jcmm.71081)
Supplement: Supplementary file 1 — Figure S1: Construct stable cell lines and explore the influence on the biological process of HCC cells. (A) Fluorescence microscopy of pLV‐NC and pLV‐HBx lentiviral cells transfected HepG2 with fluorescence microscopy. (B) Expression of HBx protein in HepG2 cells was detected by western imprinting. (C) Fluorescence expression of pLV‐NC and pLV‐shHBx lentiviral cells transfected with PLC/PRF5 was observed by fluorescence microscopy. (D) Expression of HBx protein in PLC/PRF/5 cells was detected using Western blotting. The error line represents ± standard deviation evaluated using the t‐test, and the scale length was 200 μm. ns means p > 0.05; *p < 0.05, **p < 0.01, ****p < 0.0001. The scale length was 200 μm. These results are represent of three repeated experiments. [file JCMM-30-e71081-s001.zip › Supplement the descriptions of Figure S1 with the results.docx]

**Supplement the descriptions of Figure S1 with the results**

To observe the effect of HBx on the expression of AMDM10 in HCC cells, in this study, human HCC cells HepG2 without HBV infection were transfected with a vector expressing HBx (HepG2-HBx), while human HCC cells PLC/PRF/5 carrying HBV had HBx expression knocked down (PLC/PRF/5-shHBx). These engineered human HCC cells were validated to check whether HBx could be successfully overexpressed or its expression inhibited. The results showed that these cells were successfully transfected with the overexpression vector and the knockdown vector (Figure S[1A,C](#F1)). Western blotting verification of HBx expression showed that high HBx expression in HepG2 cells (Figure S1B) and effective knockdown of HBx expression in PLC/PRF/5 cells (Figure S[1D](#F1)), indicating that these engineered human HCC cells can be used for related studies analysing the effect of HBx on AMDM10 expression in HCC cells.
