# Supplementary figures and images for: HBx Promotes Liver Cancer Cells to Escape NK‐92 Cell Attack by Mediating ADAM10 to Enzyme Cut MICA/B Shedding From Cancer Cell Membrane
Source: J Cell Mol Med. 2026 Mar 6;30(5):e71081. doi: 10.1111/jcmm.71081 (PMC12965905; doi:10.1111/jcmm.71081)

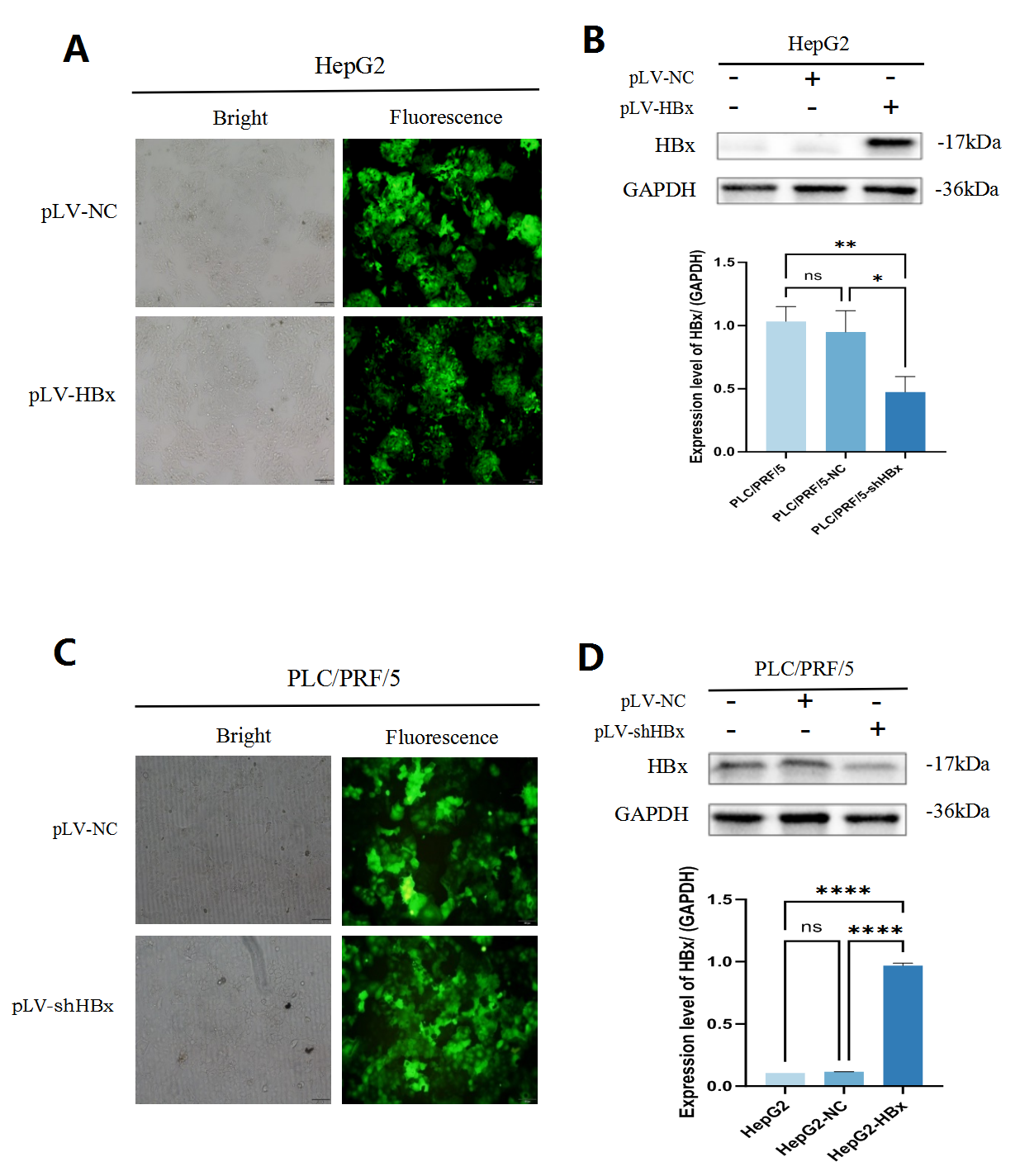

Supplement: Supplementary file 1 — Figure S1: Construct stable cell lines and explore the influence on the biological process of HCC cells. (A) Fluorescence microscopy of pLV‐NC and pLV‐HBx lentiviral cells transfected HepG2 with fluorescence microscopy. (B) Expression of HBx protein in HepG2 cells was detected by western imprinting. (C) Fluorescence expression of pLV‐NC and pLV‐shHBx lentiviral cells transfected with PLC/PRF5 was observed by fluorescence microscopy. (D) Expression of HBx protein in PLC/PRF/5 cells was detected using Western blotting. The error line represents ± standard deviation evaluated using the t‐test, and the scale length was 200 μm. ns means p > 0.05; *p < 0.05, **p < 0.01, ****p < 0.0001. The scale length was 200 μm. These results are represent of three repeated experiments. [file JCMM-30-e71081-s001.zip › jcmm71081-sup-0001-FigureS1.png]
