# Supplementary material for: HBx Promotes Liver Cancer Cells to Escape NK‐92 Cell Attack by Mediating ADAM10 to Enzyme Cut MICA/B Shedding From Cancer Cell Membrane
Source: J Cell Mol Med. 2026 Mar 6;30(5):e71081. doi: 10.1111/jcmm.71081 (PMC12965905; doi:10.1111/jcmm.71081)
Supplement: Supplementary file 2 — Videos S1–S2: High throughput intelligent living cells instrument was applied to observe the attack activity of NK‐92 cells to HepG2 cells. Video S1: HepG2 cells transfected with the control vector and co‐cultured with NK‐92; Video S2: HepG2 cells transfected with HBx overexpression vectors and co‐cultured with NK‐92 cells. The effect of NK‐92 on HepG2 cells was observed for 12 h using a high‐throughput intelligent living‐cell instrument. These results are represent of three repeated experiments. Video S1: http://video.cqdbc.org.cn/mp4/0422/1/. Video S2: http://video.cqdbc.org.cn/mp4/0422/2/. [file JCMM-30-e71081-s002.zip › Supplement the descriptions of Videos S1 and S2 with the results.docx]

**Supplement the descriptions of Videos S1 and S2 with the results**

Dynamic monitoring videos are shown as Videos S[1](#F2) and S[2](#F2). Liver cancer cells and liver cancer cells stably transfected with overexpressed HBx (OE-HepG2) vectors or small hairpin-interfered HBx (PLC/RPF/5-shHBx) vectors were co-cultured with or without NK-92 cells in a 6-well plate for 24 h, and then stained with crystal violet to observe the effect of NK-92 cells on the clonal formation of HCC cells. The results showed that HepG2 cells overexpressing HBx can significantly escape attack by NK-92 cells, while PLC/RPF/5 cells with HBx expression knocked down are more easily attacked by NK-92 cells. These findings suggested that after HCC cells are infected with HBV, the HBx protein produced by HBV promotes the escape of HCC cells from NK cell attack.
